# The Depletion of ABI3BP by MicroRNA-183 Promotes the Development of Esophageal Carcinoma

**DOI:** 10.1155/2020/3420946

**Published:** 2020-07-26

**Authors:** Hongfei Cai, Yang Li, Da Qin, Rui Wang, Ze Tang, Tianyu Lu, Youbin Cui

**Affiliations:** Department of Thoracic Surgery, The First Hospital of Jilin University, 71 Xinmin Street, Changchun 130021, China

## Abstract

**Background:**

Esophageal cancer (EC), as a serious threat to human life and health, is one of the most common cancers around the world. Many studies have suggested that many microRNAs are involved in tumorigenesis and progression.

**Methods:**

To search for a novel and promising predictive therapeutic target or biomarker to achieve the goal of the early diagnosis and treatment of EC, we used the EC cell lines Eca-109 and KYSE-150 and normal human esophageal epithelial cells (HEECs) to investigate the effect of ABI3BP on EC.

**Results:**

We found that ABI family member 3 binding protein (ABI3BP) was downregulated in EC and suppressed the proliferation, activity, migration, and invasion of EC cells. ABI3BP was downregulated by miR-183, which plays the role of an oncogene.

**Conclusion:**

ABI3BP and miR-183 can be considered potential biomarkers for the diagnosis of patients with EC and can be effective targets for antitumor therapy.

## 1. Introduction

Esophageal cancer (EC) is one of the most common malignancies and ranks eighth in prevalence throughout the world. It also remains the sixth leading cause of death due to carcinoma. EC, because of it high malignancy potential, poor prognosis, and high mortality, is one of the most serious gastrointestinal malignancies. It has been shown that its global incidence has increased sharply by more than 6-fold. The number of new cases of EC was more than 572,000 in 2018 and nearly 440,000 deaths occurred. EC poses a serious threat to human life and health worldwide [1–5]. In the past few years, with the rapid progress in surgery, chemotherapy, and preoperative radiotherapy, the survival rate has been effectively improved to some extent in patients with EC. Unfortunately, because the early symptoms of EC are not apparent, an increasing number of patients with EC are diagnosed when they are at an advanced stage and the tumor cannot be removed surgically. In addition, the poor prognosis of patients with EC is seriously affected by resistance to chemotherapy and radiotherapy as well as tumor metastasis and recurrence [6, 7]. Recent studies have reported that the 5-year survival rate and the median survival time of patients with EC are 15-20% and 1.5 years, respectively [7, 8]. Although some new treatments, such as systemic treatment, nivolumab, novel targeted therapies, and immune checkpoint inhibition, have been used recently, there are still many limitations [7, 9–12]. Therefore, it is urgent to develop new treatment methods to improve the prognosis of EC patients.

An increasing number of studies have indicated that miRNAs (miRs) play a crucial role in tumorigenesis and progression and are useful and important diagnostic and prognostic markers in human cancers [13–17]. A great number of miRNAs are also involved in the development and progression of EC. For instance, miR-377 has been proven to be significantly downregulated in both the serum and tumor tissues of patients with esophageal squamous cell carcinoma (ESCC) and was shown to be located in the chromosome 14q32 region. Moreover, the survival rate of patients is positively correlated with the expression level of miR-377. miR-377 inhibited the occurrence, growth, angiogenesis, and metastasis of ESCC by targeting the expression of CD133 and VEGF, thus acting as a tumor suppressor [18]. miR-873 has a crucial effect on the growth and metastasis of EC by targeting the DEC2 gene [19]. miR-183 was found to be involved in the pathogenesis of ESCC [20]. The above studies revealed the regulatory role of miRNAs in the occurrence, growth, and metastasis of EC and their roles as tumor suppressors or tumor promoters.

ABI3BP is an ArgBP/E3B1/Avi2/NESH family protein that is well known to be involved in the negative regulation of cell movement and metastasis through its effects on membrane folding and lamellar formation and was proven to be an SRC homologous (SH3) adaptor molecule [21, 22]. ABI3BP was considered to have a tumor-suppressive effect in thyroid cancer [23]. The online Gene Expression Profiling Interactive Analysis [24] database showed that the expression level of ABI3BP may be negatively associated with EC, while the miR-183-3p expression level may be positively correlated with EC. Moreover, ABI3BP is a potential target gene of miR-183. Taken together, the evidence suggests that ABI3BP restoration could exert inhibitory effects on EC cell growth and play a crucial role in mediating the pathogenesis, development, and/or progression of EC, indicating the possible treatment of EC by inhibiting miR-183 or enhancing ABI3BP.

## 2. Materials and Methods

### 2.1. Cell Culture

Eca-109 (KG189, KeyGEN Bio, Jiangsu, China), KYSE-150 (KG532, KeyGEN Bio, Jiangsu, China), and human normal esophageal epithelial cells (KG161, KeyGEN Bio, Jiangsu, China) were cultured in DMEM which containing 10% FBS, penicillin (100 U/ml), and streptolysin (0.1 mg/ml) in a cell incubator (5% CO_2_, 37°C). When the cells entered the logarithmic growth stage and the cell density reaches 80%~90%, the culture medium was removed, washing by PBS (10 ml) for three times, in addition to trypsin (0.25%), and was used for digestion in 37°C for 1 min. After the cells became round, fresh culture medium was added to stop digestion, and the pipette was used for repeated blowing and beating to form single-cell suspension. Then, cells were centrifuged with 2000 rpm for 2 min and used for follow-up research studies.

### 2.2. Cell Transfection

Cells were inoculated in a 6-well plate for culture. For the cells grown to 80%, the culture medium was replaced with a nonantibiotic culture medium 2 hours before transfection for further transfection. Prepare the transfection complex; dissolve the siRNA oligomer (2.5 g) or plasmid (2.5 g) into 250 living room culture solution free of serum and antibiotics. The culture medium with no serum and no antibiotics was diluted to 250 living room Lipofectamine 2000 and incubated for five minutes at room temperature (RT).The above diluted Lipofectamine 2000 and siRNA/plasmid were mixed and incubated at RT for twenty minutes. Add the above mixture (500 livl/hole) to the incubator plate for 6 hours. The cells were replaced with a complete culture medium and continued for 24 hours/48 hours for subsequent research studies [25, 26].

### 2.3. CCK8 Assay

After 24 h cell transfection, trypsin digested the cells to prepare cell suspension. These cells were cultured in 96-well plates in an incubator at a thickness of 2000/well. When cultivating for 0, 24, 48, and 72 h, respectively, add 10 living room CCK8 reagent to each well of the 96-well plate, and continue to cultivate for 1 hour. After the incubation, the absorbance value of OD450 nm in each well was detected with an enzyme marker, and the cell proliferation curve was plotted according to the absorbance value [27, 28].

### 2.4. Transwell Invasion Assay

Matrigel was diluted with serum-free precooling medium and placed at the bottom of the upper chamber of the Transwell chamber for incubation at 37°C for five hours. The basement membrane was rehydrated with the culture medium which is serum-free. Twenty-four hours later after transfection, trypsin was used to digest the cells and serum-free culture medium was preparing cell suspension. Take out the Transwell chamber, and wipe the remaining cells in the chamber by cotton swabs after incubation during all night.

Wash these cells by PBS for three times, and then fix with 4% paraformaldehyde for thirty minutes. After that, the cells were treated with 0.1% crystal violet for 20 min. Then, wash it by PBS for three times, and select randomly for three fields for counting under the microscope (100x).

### 2.5. Flow Cytometry Assay

Twenty-four hours later after transfection with the cells, replace the culture medium by the serum-free medium to culture for twenty-four hours. The cells were cultured followed by trypsin digestion fluid without EDTA and collected by centrifugation. The density of cell was controlled to 1‐5 × 10^6^/ml. Using a pipette, 100 living room cell suspension was absorbed into the flow tube. After adding 5 living room cells Annexin V/FITC, cultivate it out of light for five minutes at RT. Add 10 livl PI dye solution and 400 livl PBS to the flow tube, and then conduct apoptosis detection on the flow cytometer. The data obtained were analyzed using FlowJo software.

### 2.6. Wound Healing Assay

After 24 h cell transfection, trypsin was used to digest the cells to prepare cell suspension as described above. Then, cells were cultured at 37°C for twelve hours in 6-well plates, and the cell density was controlled to about 5 × 10^5^ per well. The sterilized suction head was used to cut a wound in the middle of each hole of the 6-hole plate, and the photos were taken after PBS washing for 2 times (Nikon Eclipse Ti-S, 20x). Culture was continued for 24 h, and the wound was photographed again. Five photos were taken for each well. ImageJ software was used to image the wounds taken twice before and after, and the wound closure was measured.

### 2.7. Dual-Luciferase Reporter Assay

Mouse *ABI3BP* mRNA and miR-183-3p were synthesize by GENEWIZ (Suzhou, China) and subcloned in pmirGLO and pCMV plasmids. Dual-luciferase reporter assay kit was purchased from Beyotime Biotechnology (Shanghai, China).

The cells were cultured as described above. After digested into single-cell suspension, the cells were divided into 2 groups and cultured in a 24-well plate. Transfection was carried out when the cell density reached about 70-80%. The cells were transfected with pmirGLO-ABI3BP+pCMV-NC and pmirGLO-ABI3BP+pCMV-miR-183-3p, respectively, by using Lipofectamine™ 3000 Transfection Reagent (L3000001, Thermo Fisher). Then, the cells were cultured for 48 hours in normal condition. Then, the cells were collected and the fluorescence values of the cells in each group were immediately determined according to the protocol provided in the kit.

### 2.8. Statistical Analysis

Three independent repeated experiments were carried out in this study. The experimental data were expressed as mean ± SEMusing GraphPad Prism 7.0 software for analyzing. Student's *t*-test or single-factor ANOVA was used to analyze the differences between groups, and the value of *P* less than 0.05 was considered statistically significant.

## 3. Results

### 3.1. ABI3BP and miR-183 Are Related to EC

**Figure 1:**
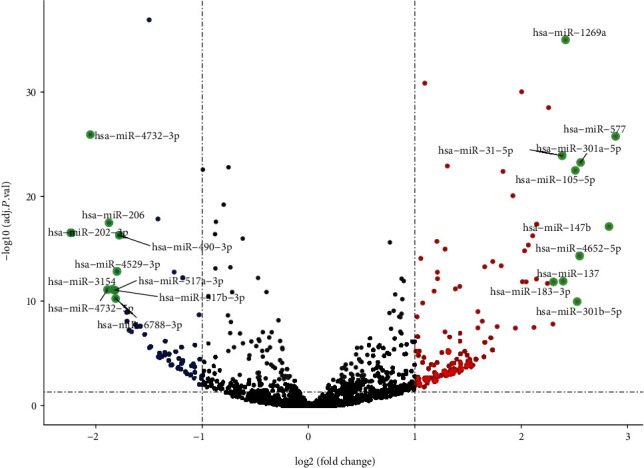
The expression of miRNAs in EC tissues and normal tissues from GEPIA analysis. The top 10 variation miRNAs were marked in the map.

**Figure 2:**
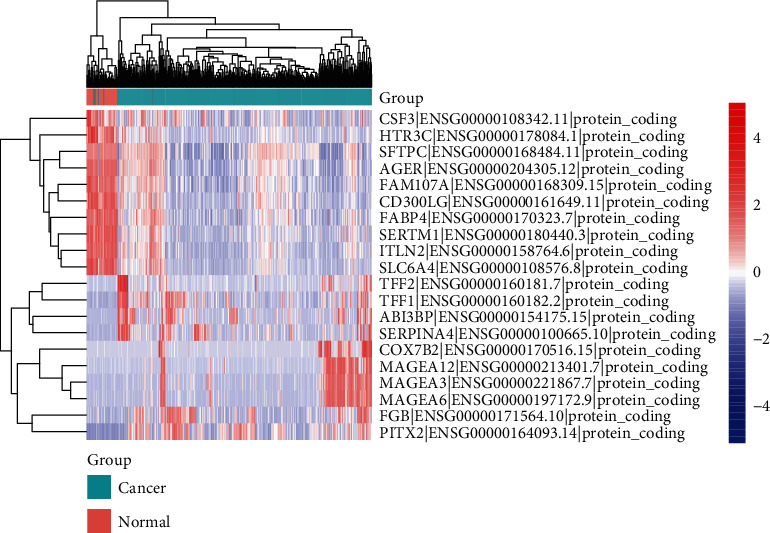
The expression of mRNAs in EC tissues and normal tissues from GEPIA analysis. The top 10 variation mRNAs were showed in the heat map.

Through the online Gene Expression Profiling Interactive Analysis database, our team analyzed public data and found that the ABI3BP expression level may be negatively correlated with EC, while the miR-183-3p expression level may be positively correlated with EC (Figures 1 and 2). According to previous studies, miR-183 participates in the pathogenesis of ESCC [20], while ABI3BP is related to gallbladder cancer, thyroid tumors, and lung cancer [29–32]. These results strongly suggest that ABI3BP and miR-183 could regulate EC development.

**Figure 3:**
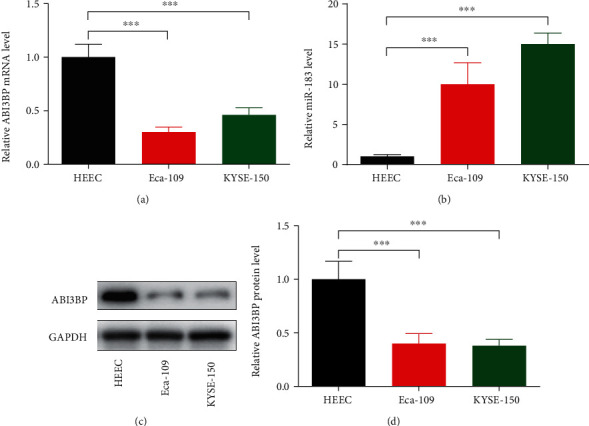
The expression levels of ABI3BP and miR-183 in HEEC, Eca-109, and KYSE-150 cells. Three kinds of cells were cultured and harvested when growing to 80-90%. Total mRNA or protein was extracted and detected by real-time PCR or Western blot analysis. (a) Relative ABI3BP mRNA level was detected by using RT-PCR. Five independent experiments (*n* = 5) were repeated for each cell line. (b) Relative miR-183-3p level was detected by using RT-PCR. Five independent experiments (*n* = 5) were repeated for each cell line. (c) Relative ABI3BP protein level was detected by Western blot. Five independent experiments (*n* = 5) were repeated for each cell line. (d) Quantitative analysis of (c). ^∗∗∗^*P* < 0.001, one-way ANOVA with post hoc.

Then, the mRNA expression levels of ABI3BP and miR-183 in KYSE-150 and Eca-109 cells and HEECs were detected by RT-PCR. The results revealed that, compared with those in HEECs, the expression levels of ABI3BP were markedly downregulated in KYSE-150 and Eca-109 cells (Figure 3(a)); furthermore, miR-183 was significantly upregulated (Figure 3(b)), suggesting that the expression of ABI3BP and miR-183 were altered in ECs. The protein level of ABI3BP was also detected in the three cell lines, which showed that ABI3BP was significantly downregulated in KYSE-150 and Eca-109 cells (Figures 3(c) and 3(d)).

### 3.2. ABI3BP Is a Target of miR-183-3p in EC Cells

**Figure 4:**
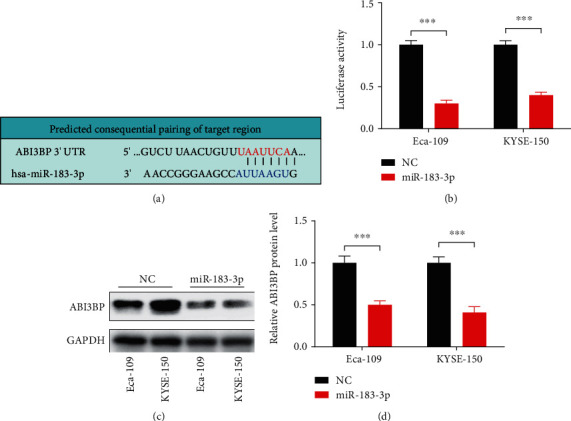
ABI3BP may be one of the targets of miR-183 in Eca-109 and KYSE-150 cells. (a) Predicted consequential pairing site of ABI3BP mRNA and miR-183-3p. (b) Dual-luciferase reporter assay was performed to detect the luciferase activity of ABI3BP mRNA and miR-183-3p. Three independent experiments (*n* = 3) were repeated. (c) Two kinds of cells were cultured and harvested when growing to 80-90%. Total protein was extracted and detected by Western blot analysis. Relative ABI3BP protein level was detected by Western blot under miR-183-3p overexpression condition. Five independent experiments (*n* = 5) were repeated for each cell line. (d) Quantitative analysis of (c). ^∗∗∗^*P* < 0.001, Student's *t*-test.

The potential target gene of miR-183-3p was predicted using an online bioinformatics database (TargetScan). Our team found that ABI3BP contains binding sites that might interact with miR-183-3p and may possibly be a target gene of miR-183-3p (Figure 4(a)). To further verify the above hypothesis, we constructed a dual-luciferase reporter gene and transfected it into Eca-109 cells. The results suggested that miR-183-3p markedly decreased the luciferase activity of ABI3BP (Figure 4(b)). Therefore, miR-183-3p can bind to ABI3BP, which may be its target gene. To further verify this result, we transfected miR-183-3p mimics into the cells and detected the expression levels of ABI3BP in Eca-109 cells. The result suggested that the expression levels of ABI3BP protein were markedly reduced after transfection (Figures 4(c) and 4(d)), which further proved that ABI3BP was a target of miR-183-3p.

### 3.3. ABI3BP and miR-183 Modulate the Apoptosis and Proliferation of EC Cells

**Figure 5:**
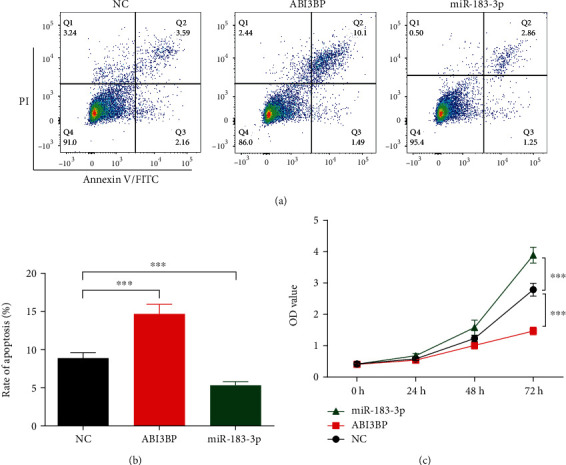
ABI3BP and miR-183 modulate the apoptosis and proliferation in EC cells. (a) After being transfected with ABI3BP or miR-183 for 24 h, flow cytometry assay was performed to detect the rate of apoptosis in Eca-109 cells under ABI3BP or miR-183 overexpression conditions. Three independent experiments (*n* = 3) were repeated for each group. (b) Quantitative analysis of Figure 5(a). (c) After being transfected with ABI3BP or miR-183 for 24 h, CCK8 assay was performed to assess the proliferation of Eca-109 under ABI3BP or miR-183 overexpression conditions. *n* = 5 for each group. ^∗∗∗^*P* < 0.001, one-way ANOVA with post hoc and two-way ANOVA with post hoc.

To further explore the functions of ABI3BP and miR-183 in EC, we designed experiments to detect the apoptosis and proliferation of Eca-109 cells under ABI3BP or miR-183 overexpression conditions. The flow cytometry assay revealed that ABI3BP could obviously increase the rate of apoptosis, while miR-183 could significantly reduce the rate of apoptosis (Figures 5(a) and 5(b)), which suggested that ABI3BP plays a role as a tumor suppressor and that miR-183 is an oncogene. The CCK8 assay indicated that the viability of Eca-109 cells was markedly decreased by ABI3BP and increased by miR-183 (Figure 5(c)). All these results are consistent with the database results (Figures 1 and 2).

### 3.4. ABI3BP and miR-183 Modulate the Migration and Invasion of EC Cells

**Figure 6:**
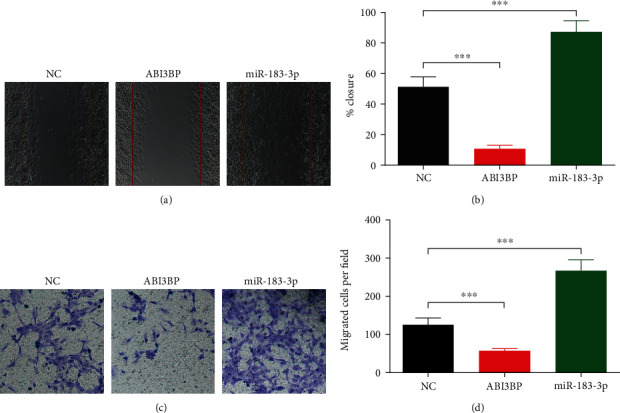
ABI3BP and miR-183 modulate the migration and invasion in EC cells. (a) After being transfected with ABI3BP or miR-183 for 24 h, the migration ability of EC cells was assessed by wound healing assay. Five independent experiments (*n* = 5) were repeated for each group. (b) Quantitative analysis of (a). (c) After being transfected with ABI3BP or miR-183 for 24 h, the invasion ability of EC cells was assessed by Transwell assays. Five independent experiments (*n* = 5) were repeated for each group. (d) Quantitative analysis of (b). ^∗∗∗^*P* < 0.001, one-way ANOVA with post hoc.

Next, we detected the invasion and migration of EC cells under ABI3BP or miR-183 overexpression conditions. In the wound healing assay, ABI3BP markedly reduced the migration of EC cells, while miR-183 markedly promoted EC cell migration (Figures 6(a) and 6(b)). Additionally, the Transwell invasion assay showed that ABI3BP could significantly inhibit the invasion of EC cells, while miR-183 promoted EC cell invasion (Figures 6(c) and 6(d)).

## 4. Discussion

Despite the rapid progress in surgery, chemotherapy, and preoperative radiotherapy techniques for EC made in recent years, unfortunately, an increasing number of patients with EC are diagnosed when they are at an advanced stage and the tumor cannot be removed surgically because the early symptoms of EC are not obvious. In addition, the poor prognosis of patients with EC is seriously affected by resistance to chemotherapy and radiotherapy as well as tumor metastasis and recurrence. Numerous lncRNAs and microRNAs have been proven to play a crucial role in tumor development and drug resistance and can be used as potential biomarkers for tumor diagnosis and prognosis and as molecular therapeutic targets [33–35].

Human ABI3BP was originally identified as a novel NESH-binding protein that is involved in tumor cell migration and mobility [36]. Through its interaction with NESH, ABI3BP may exert a great effect on the progression of tumor cells. In fact, the expression level of ABI3BP mRNA was markedly decreased in human lung cancer cell lines and clinical samples. Although the molecular mechanisms of tumorigenesis and progression are not well understood, the progression of tumorigenesis is closely related to the cell cycle. For example, p53, RB, or p19ARF, all of which are tumor suppressor proteins, play important roles in the cell cycle. It has been reported that the expression of ABI3BP is downregulated in gallbladder cancer tissues, and ABI3BP is a downstream target of metastasis-associated lung adenocarcinoma transcript 1 [29]. Studies have confirmed the tumor-suppressive effect of ABI3BP, suggesting that the ectopic expression of ABI3BP can impair the growth characteristics of cancer cells and induce senescence [23]. Depletion of ABI3BP could induce oncogenic transformation [37]. ABI3BP recovery was highlighted as an important factor associated with thyroid tumors that inhibited tumor growth and invasion and promoted cell senescence [32]. In addition, ABI3BP was reported to show decreased expression in lung cancer cell lines, suggesting that ABI3BP could be a biomarker for lung cancer progression [31].

Here, in our study, we found that there is a low level of ABI3BP expression in EC cells and a high level of miR-183 expression. Additionally, we found that ABI3BP is one of the targets of miR-183. Together, the results indicated that miR-183 reduced ABI3BP protein levels and thus promoted EC development by enhancing cell proliferation, migration, and invasion and inhibiting apoptosis.

We showed for the first time that ABI3BP is downregulated in EC, revealing the role of ABI3BP as a tumor suppressor gene in the progression of EC growth and metastasis at the cellular level. It was further proven that miR-183 is an upstream regulatory molecule of ABI3BP. The molecular mechanism of the promotion by miR-183 of the growth and metastasis of EC cells mediated by regulating ABI3BP was demonstrated. In our future work, the mechanism of ABI3BP involved in its tumor-suppressive effects will be studied in detail, and ABI3BP will be explored as an effective target for tumor diagnosis and as a biomarker.

## 5. Conclusions

ABI3BP plays the role of a tumor suppressor gene in the progression of EC growth and metastasis at the cellular level. miR-183 is an upstream regulatory molecule of ABI3BP. ABI3BP and miR-183 can be considered potential biomarkers for the diagnosis and prediction of the progression and prognosis of EC patients and can be effective targets for antitumor therapy.

## Data Availability

The data in the manuscript are original and available.
